# Epigenetic histone modifications in kidney disease and epigenetic memory

**DOI:** 10.1007/s10157-025-02668-x

**Published:** 2025-04-05

**Authors:** Kensuke Sasaki, Takao Masaki

**Affiliations:** Department of Nephrology, Hiroshima University Hospital, 1-2-3 Kasumi, Minami-Ku, Hiroshima, 734-8551 Japan

**Keywords:** Epigenetics, Renal fibrosis, Diabetic kidney disease, Chronic kidney disease, Hypertension, Epigenetic memory

## Abstract

**Background:**

Epigenetic mechanisms, including DNA methylation, histone modifications, and non-coding RNAs, are influenced by environmental factors and play a central role in the progression and therapeutic targeting of kidney diseases, such as diabetic kidney disease (DKD), chronic kidney disease (CKD), and hypertension. These epigenetic changes are also preserved as cellular memory, with this “epigenetic memory” known to have long-term effects on such chronic diseases.

**Summary:**

Histone modifications are readily reversible epigenetic changes that regulate gene expression by altering chromatin structure or providing docking sites for transcriptional regulators. From a disease perspective, the involvement of epigenetics and “epigenetic memory” in DKD, CKD, senescence, and hypertension has been increasingly studied in recent years. Targeting epigenetic mechanisms is, thus, expected to offer novel therapeutic strategies for these diseases. Advances in treatment include histone deacetylase inhibitors and methyltransferase inhibitors, their applications of which have expanded from oncology to nephrology. However, challenges such as long-term toxicity and off-target effects remain significant. Further elucidation of kidney-specific epigenetic pathways and memory mechanisms may pave the way for precision epigenetic therapies, enabling the reversal of pathological epigenetic signatures and the mitigation of disease progression.

**Conclusion:**

This review integrates recent advancements, highlighting functional evidence that histone modifications, particularly histone tail methylation, are involved in the pathogenesis of kidney diseases. It also emphasizes the translational significance of these findings, underlining the potential of epigenetics-based therapies to transform the management of kidney diseases.

## Introduction

**Fig. 1:**
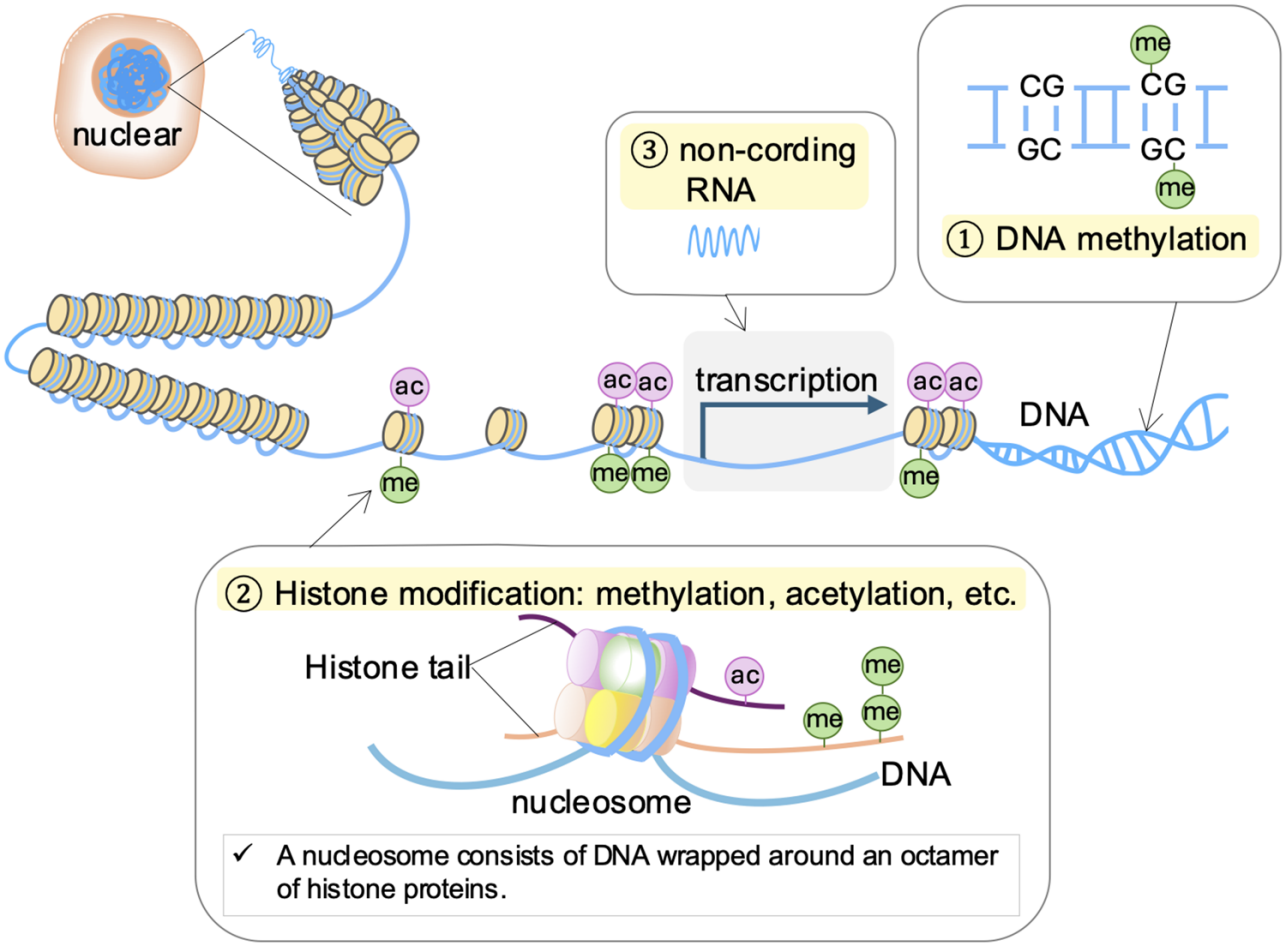
Overview of epigenetic modifications. Chromosomes consist of a complex of DNA and proteins known as chromatin. The fundamental subunit of chromatin is the nucleosome, which is composed of an octamer of core histone proteins (H2A, H2B, H3, and H4) wrapped by DNA. Beyond serving as a structure essential for compacting vast amounts of genetic information, nucleosomes act as a natural barrier to polymerases that require access to DNA

**Fig. 2:**
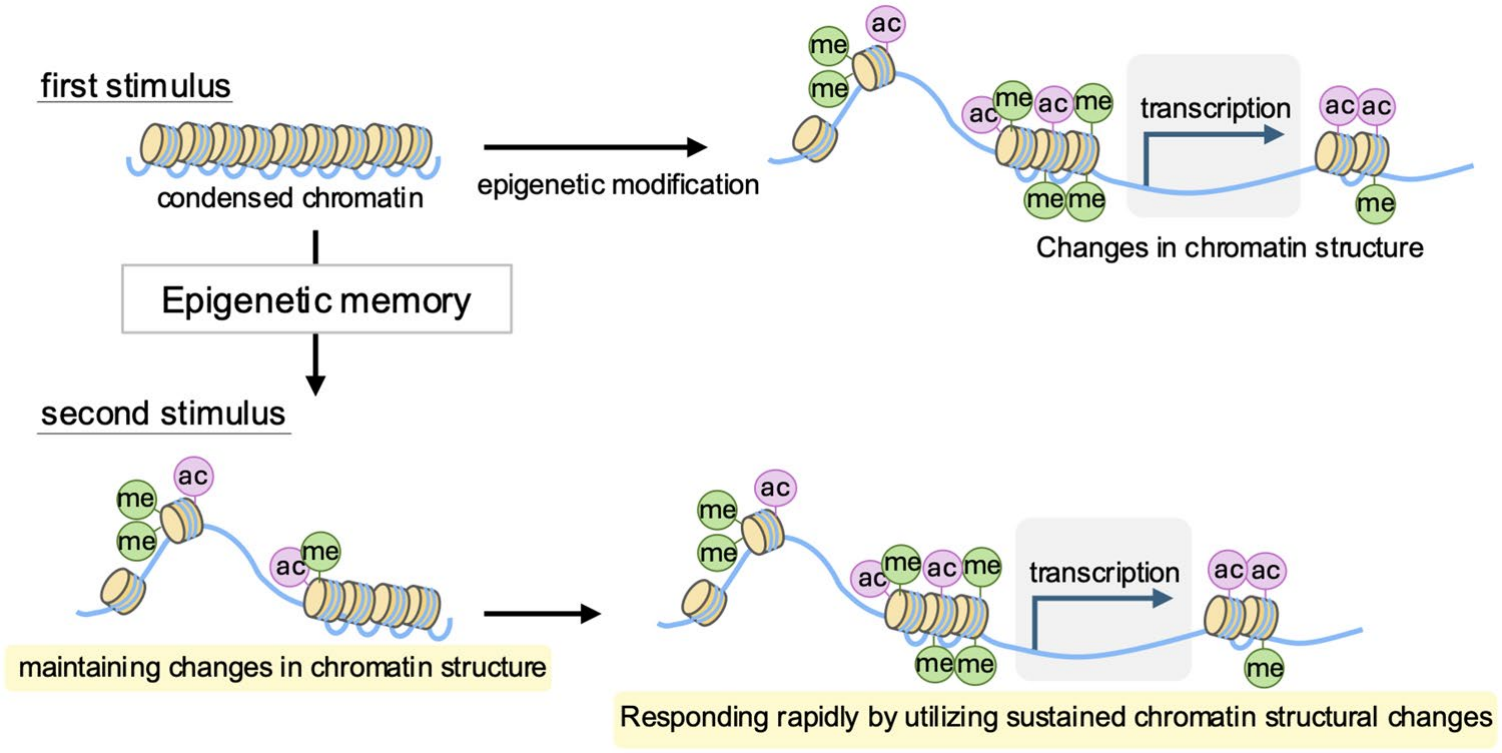
Concept of epigenetic memory. Initial stimuli induce changes in histone structure. These changes are stored as epigenetic memory, which maintains the epigenetic state in response to the original stimulus. Upon a second stimulus, cells with epigenetic memory can respond rapidly and effectively to the trigger

Epigenetics refers to mechanisms of gene expression regulation that do not involve changes to the DNA sequence. These mechanisms include reversible modifications to DNA and histones, which alter chromatin structure and influence gene expression through transcriptional and post-transcriptional processes [1]. Key epigenetic mechanisms include DNA methylation, histone modifications, and non-coding RNAs [2] (Fig. 1). Information conveyed through epigenetic modifications can be influenced by environmental factors [2], potentially contributing to complex, multifactorial diseases such as diabetes, hypertension, and chronic kidney disease [3–5]. Moreover, epigenetic modifications are preserved as cellular memory, with this “epigenetic memory” exerting long-term effects on chronic conditions [6–8]. For instance, initial stimuli, such as high glucose levels, can induce histone modifications and changes in chromatin structure, which are stored as epigenetic memory within the cell [3, 7]. Upon subsequent stimulation, this memory enables a rapid response through the enhanced expression of downstream genes facilitated by transcription factor binding (Fig. 2). However, in the context of disease, epigenetic memory is also thought to contribute to treatment resistance.

In the field of nephrology, substantial information is available on DNA methylation and histone modifications [9]. DNA methylation is a relatively stable and heritable epigenetic mark [1], whereas histone modifications represent more dynamic and reversible epigenetic changes [10], regulated by specific enzymes, particularly in the case of histone tail methylation [11]. In this comprehensive review, we aim to provide functional evidence that emphasizes the role of histone modifications—specifically histone tail methylation—in the development of kidney diseases. Non-coding RNAs, including miRNAs, have been extensively reviewed elsewhere [12] and are beyond the scope of this review.

## Histone modifications

Histones, including core histones (H2A, H2B, H3, H4) and linker histones (H1, H5), are highly conserved, positively charged basic proteins essential for DNA packaging. These proteins interact with negatively charged DNA through electrostatic forces, enabling DNA to be compactly organized into a highly condensed and orderly chromatin unit known as the nucleosome [13]. Each nucleosome consists of DNA wrapping around a core histone octamer, which includes two molecules each of H2A and H2B dimers, and a single H3-H4 tetramer [14]. Histone modifications encompass a variety of covalent post-translational modifications on core histone proteins, including acetylation, methylation, phosphorylation, ubiquitination, SUMOylation, citrullination, biotinylation, crotonylation, and ADP-ribosylation [15]. These modifications primarily occur at the amino termini of histones, where they modulate chromatin structure and provide docking sites for transcriptional regulators, thereby influencing gene expression either positively or negatively [14]. Histone modification factors are categorized into “writers,” such as histone acetyltransferases and methyltransferases; “erasers,” such as deacetylases and demethylases; and “readers,” which, although not directly modifying the epigenetic code, play a critical role in interpreting and stabilizing epigenetic information and recruiting effector proteins to specific loci [1]. Furthermore, histone marks contribute to the functional division of chromatin into silent heterochromatin and active euchromatin, and they play essential roles in chromosome condensation and higher order chromatin organization [16].

### Histone acetylation

Histone acetylation, one of the most extensively studied histone modifications, is mediated by histone acetyltransferases (HATs), which transfer negatively charged acetyl groups (COCH₃) to lysine residues on core histones. By neutralizing the positive charge on lysine, histone acetylation promotes an open chromatin structure, facilitating access for transcription factors and thereby enhancing transcription [17]. In addition, acetylated histone residues serve as docking sites for transcriptional coactivators, further promoting transcription [18]. Histone acetylation is reversible, with deacetylation catalyzed by histone deacetylases (HDACs), which are classified into four classes: Class I (HDAC1, 2, 3, 8), Class II (HDAC4, 5, 6, 7, 9, 10), Class III (sirtuins, SIRT1–7), and Class IV (HDAC11). Class I HDACs are ubiquitously expressed in the nucleus and are essential for cell survival and proliferation, with HDAC8 also localized to the cytoplasm and associated with the cell membrane. Class II HDACs, further subdivided into Class IIa and IIb, may have tissue-specific roles and are localized to both the nucleus and the cytoplasm [19]. Class III HDACs, consisting of the sirtuins (SIRT1 to SIRT7 in mammals), rely on NAD⁺ for catalytic activity, distinguishing them from the “classical” HDACs in Classes I, II, and IV, which require zinc for their activity [20]. HDAC11 is the sole member of Class IV. Each HDAC class and subtype possesses distinct substrates, cellular localizations, and biological functions, playing pivotal roles in regulating gene expression and diverse cellular processes.

### Histone methylation

Histone methylation is the process by which methyl groups (CH_3_) are added to the lysine or arginine residues of core histones, using S-adenosyl-L-methionine as a methyl donor. This process is catalyzed by histone methyltransferases (HMTs), such as protein arginine methyltransferases (PRMTs) and lysine methyltransferases (KMTs). Both lysine and arginine can undergo multiple methylation events on their side chains. Specifically, lysine residues can be monomethylated, dimethylated, or trimethylated on their ε-amino group (e.g., H3K4me2, H3K4me3), while arginine can be monomethylated or dimethylated symmetrically or asymmetrically on its guanidino group [21]. Unlike histone acetylation, which alters the charge of histone proteins, histone methylation serves as a docking platform for transcription factors. The functional outcome of histone methylation—whether it promotes or represses transcription—depends on the specific amino acid residue modified and the degree of methylation. For instance, methylation at H3 lysine 4 (H3K4), H3K36, and H3K79 is generally associated with transcriptional activation, whereas methylation at H3K9, H3K27, and H4K20 is linked to transcriptional repression [22]. Methyl groups can be removed by histone demethylases, such as amine oxidase–lysine-specific demethylase (LSD1, also known as KDM1A) [23]. In addition, protein arginine deiminases (PADs or PADIs) can convert arginine and monomethylated arginine residues into citrulline [24]. Histone methylation creates a versatile platform for transcription factor binding and, depending on the residue, methylation state, and biological context, can either facilitate or inhibit gene expression [25, 26].

### Other histone modifications

In addition to the modifications mentioned above, histone phosphorylation, ubiquitylation, and glycosylation have been extensively studied, with modification sites that include serine, threonine, and arginine residues [27]. Moreover, emerging modifications such as histone crotonylation and lactylation have been reported as potential contributors to kidney disease and are increasingly attracting attention [28–31].

## Histone modifications in kidney disease and epigenetic memory

### Targeting diabetic kidney disease

The onset and progression of diabetic kidney disease (DKD) involve not only genetic factors but also environmental influences, with considerable evidence highlighting the role of epigenetic mechanisms [3, 32]. In the streptozotocin-induced diabetic nephropathy mouse model, for example, the expression of SIRT1, a histone deacetylase (HDAC), is reduced. However, when SIRT1 is overexpressed specifically in renal tubules, it deacetylates and activates DNMT1, leading to DNA methylation of the *Cldn1* gene, which encodes Claudin-1, an intercellular adhesion protein in glomerular podocytes. This epigenetic modification reduces Claudin-1 expression, effectively mitigating albuminuria [33]. These findings suggest potential crosstalk between podocytes and proximal tubules mediated by epigenetic regulation.

The association between “metabolic memory,” which refers to the increased future risk of developing DKD despite subsequent normalization of blood glucose control through dietary or pharmacological interventions [34, 35], and “epigenetic memory,” which denotes the dynamic and heritable epigenetic regulation of gene expression in response to past stimuli such as hyperglycemia in diabetes, has been suggested [3, 36]. For instance, in DKD, persistent expression of DKD-related genes has been observed regardless of subsequent glycemic control [3]. Furthermore, profiling of gene expression, DNA methylation, and chromatin accessibility in renal proximal tubular epithelial cells obtained from patients with type 2 diabetes and non-diabetic individuals revealed persistent DNA methylation and chromatin changes in the proximal tubular epithelial cells of patients with type 2 diabetes [36]. However, the underlying molecular mechanisms of these changes remain unclear. Therefore, comprehensive studies on the epigenomic changes leading to persistent gene expression and the associated mechanisms may enable the development of novel therapeutic strategies aimed at erasing epigenetic memory and preventing the progression of DKD.

DNA methylation profiling of kidney biopsy samples from DKD patients has shown that methylated regions are localized within enhancer regions of transcription factors critical for renal function and are enriched with binding motifs for these transcription factors [37]. Epigenome-wide association studies (EWAS) of patient samples have proven useful for investigating epigenetic memory associated with various diseases and conditions. For example, a meta-analysis of six EWAS studies on Alzheimer’s disease (*N* = 1,453) identified differential disease-related methylation differences across various brain regions [38]. In addition, a study examining preterm infants investigated whether methylation changes related to fetal transcription persist after birth and identified numerous DNA methylation regions as potential candidates for gestational age-associated epigenetic memory [39]. Therefore, EWAS hold significant promise for advancing personalized therapeutic approaches to DKD and may offer avenues to reverse DKD-associated epigenetic memory.

### Targeting chronic kidney disease

A seminal study in the field of epigenetics and chronic kidney disease (CKD), published in 2010, demonstrated that methylation of the promoter of *Rasal1*—a gene encoding a Ras inhibitor—by the DNA methyltransferase DNMT1 activates renal fibroblasts and promotes renal fibrosis [40]. Another study revealed that the antifibrotic factor BMP7 induces demethylation mediated by TET3, a demethylase, thereby suppressing renal fibrosis [41]. Furthermore, mice with podocyte-specific deletion of the transcription factor KLF4 showed exacerbated proteinuria upon adriamycin administration. Restoring KLF4 expression reduced DNA methylation of the *nephrin* promoter,an essential slit diaphragm component, thereby ameliorating proteinuria [42].

Acute kidney injury (AKI) is not always a transient condition; even after apparent recovery, it may progress to CKD over time (AKI-to-CKD transition), ultimately leading to end-stage renal disease. Numerous studies have implicated epigenetic mechanisms in AKI, CKD, and the AKI-to-CKD transition [43–45]. A study from 2008 identified dynamic changes in histone acetylation during AKI caused by renal ischemia–reperfusion injury (IRI) [46]. Severe unilateral ischemia temporarily reduced H3 acetylation in proximal tubular cells, likely due to decreased histone acetyltransferase activity. However, upon reperfusion, H3 acetylation was restored, inducing BMP7 expression, which is essential for kidney repair, partially attributed to the selective downregulation of HDAC5 [46].

**Fig. 3:**
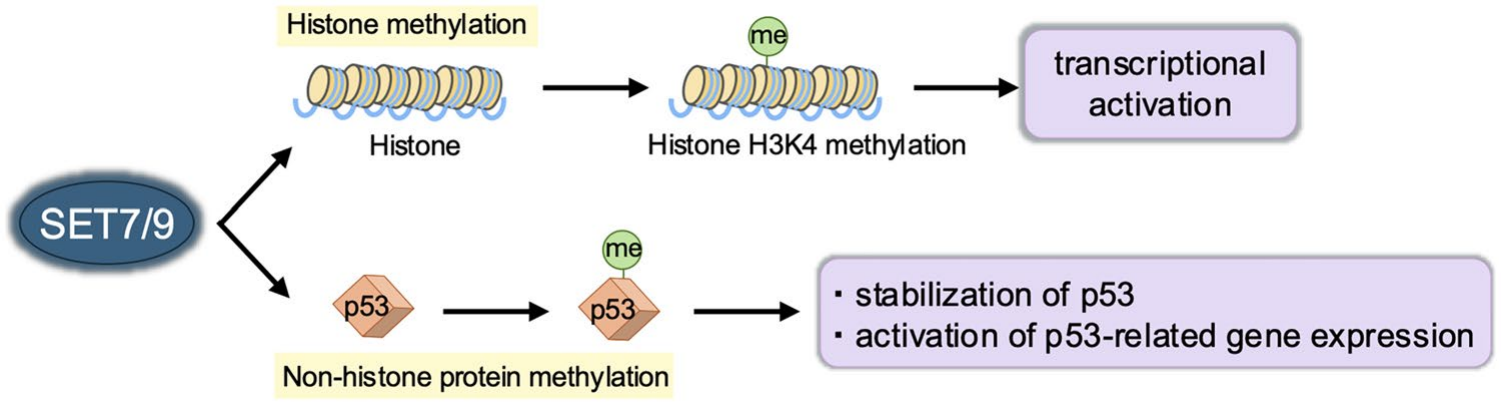
Methylation of histone and non-histone proteins by SET7/9. SET7/9 is known to methylate histone H3 at lysine 4 (H3K4). In addition, it stabilizes and activates the non-histone protein p53 through methylation of lysine 372 (K372). Furthermore, SET7/9 has been reported to methylate other non-histone proteins, such as estrogen receptor α, YAP, TAF-10, and STAT3, highlighting its diverse regulatory functions

Our research has contributed notable findings on histone methylation and renal fibrosis. The histone methyltransferase SET7/9 methylates histone H3K4 [47] and non-histone proteins such as p53 [48], estrogen receptor α [49], YAP [50], TAF-10 [51], and STAT3 [52] (Fig. 3). Recent studies have linked H3K4 methylation mediated by SET7/9 to renal disease. In the context of cancer and epigenetics, not only changes in histone modifications at specific regions but also global alterations in histone modifications have been observed [53]. In contrast, most histone modification changes identified in the field of renal failure have so far been reported to be restricted to specific gene regions. TGF-β1-induced upregulation of extracellular matrix (ECM)-related genes is associated with increased H3K4me1/2/3 and SET7/9 expression [54]. SET7/9 also promotes Smad3 activation, which drives renal fibroblast activation under TGF-β1 stimulation [55]. Using sinefungin, a selective SET7/9 inhibitor, or SET7/9-targeted siRNA, renal fibrosis was attenuated in unilateral ureteral obstruction (UUO) mice, as evidenced by decreased expression of mesenchymal markers and ECM proteins. Sinefungin also inhibited TGF-β1-induced α-smooth muscle actin expression in cultured renal epithelial cells and renal interstitial fibroblasts. In addition, SET7/9 inhibition reduced H3K4 monomethylation on promoters of fibrosis-related genes, demonstrating direct regulation by SET7/9 [5]. Notably, SET7/9 expression was found to correlate positively with the degree of interstitial fibrosis in kidneys from patients with IgA nephropathy and membranous nephropathy [5]. The histone-modifying enzyme G9a, which induces mono- and di-methylation of H3K9, a repressive chromatin marker [56, 57], also plays a role in renal fibrosis. G9a activity, induced by TGF-β1, promotes epithelial–mesenchymal transition in cancer cells [58]. Meanwhile, in a renal fibrosis mouse model, elevated G9a expression was observed. Using G9a-targeted siRNA and the specific inhibitor BIX01294, G9a inhibition reduced fibroblast activation, ECM protein accumulation, and H3K9 methylation while increasing klotho expression. In vitro, BIX01294 attenuated TGF-β1-induced fibrotic changes in renal epithelial cells and klotho downregulation, directly impacting H3K9 monomethylation at the klotho gene promoter [59]. Histone modifications encompass chemical alterations of core histones (H2A, H2B, H3, and H4) and the replacement of canonical histones by variants such as H2AX and H2AZ [60]. Our studies have evaluated the roles of the histone variant H3.3 and its chaperone, histone cell cycle regulation defective homolog A (HIRA), in UUO-induced renal fibrosis. Elevated H3.3 and HIRA levels were observed in UUO mice. Knockdown of HIRA reduced H3.3 expression and fibrosis in TGF-β1-stimulated NRK-52E cells. Chromatin immunoprecipitation analysis also revealed the co-localization of HIRA with H3.3 on promoters of activated fibrosis-related genes [61]. In a methylglyoxal-induced peritoneal fibrosis mouse model, as well as in non-adherent cells isolated from effluents of peritoneal dialysis patients, increased SET7/9 and G9a expression was noted. Inhibition of SET7/9 and G9a by sinefungin and BIX01294, respectively, reduced peritoneal collagen deposition and improved peritoneal function, accompanied by decreases in H3K4me1 and H3K9me1 [62, 63]. The roles of key histone methylation marks and their associated methyltransferases in renal fibrosis are summarized in Table 1.

**Table 1:** Roles of key histone methylation marks and their associated methyltransferases in renal fibrosis in vivo

Methyltransferase	Histone site	Intervention	Experimental model	Results	Reference
Set7/9	H3K4me1	sinefungin	UUO	Mesenchymal markers↓, ECM↓	[5]
G9a	H3K9me1	BIX01294	UUO	Mesenchymal markers↓, ECM↓, Klotho↑	[59]
EZH2	H3K27me3	3-DZNeP	UUO	Mesenchymal markers↓, ECM↓	[89]
	H3K27me3	3-DZNeP	Hyperuricemia	Mesenchymal markers↓, ECM↓	[90]
MLL1/WDR5	H3K4me3	MM-102, OICR-9429	IR induced fibrosis	Mesenchymal markers↓, ECM↓, p16↓	[77]
Dot1L	H3K79me2	EPZ5676	UUO	ECM↓, G2/M arrest↓	[91]
SMYD2	H3K36me3	AZ505	UUO	Mesenchymal markers↓, ECM↓	[92]
PRMT1	H4R3me2a	AMI-1	UUO	Mesenchymal markers↓, ECM↓	[93]

ECM, extracellular matrix

The “memory” of acute kidney injury (AKI) is thought to contribute to the progression of CKD. Renal hypoxia resulting from AKI induces the expression of inflammatory and profibrotic genes in tubular epithelial cells through epigenetic changes, known as “hypoxic memory” [45, 64]. This state activates inflammatory cells such as macrophages and neutrophils, contributing to tissue regeneration, scarring, and ultimately tubulointerstitial fibrosis [65–69]. Recently, epigenetic memory contributing to the AKI-to-CKD transition has been classified into “driving” and “priming” memory, based on the persistence of changes in gene expression control [70]. “Driving” memory, which sustains changes in gene expression, may activate fibrotic genes or inhibit renoprotective ones, thus contributing to disease progression. This process likely underlies the inflammatory and fibrotic phenotypes observed in maladaptively repaired tubular cells after injury. By contrast, “priming” memory accumulates in tubular cells that appear well repaired without detectable sustained phenotypic changes. This process may contribute to the AKI-to-CKD transition by enhancing profibrotic gene expression upon subsequent AKI episodes, creating a cumulative effect essential for wound healing [70]. Currently, we are focusing on accumulating research data on SET7/9 in relation to renal fibrosis and epigenetic memory.

### Targeting senescence

Senescence is a recognized risk factor for CKD, with epigenetic mechanisms playing a pivotal role in this process. Mutations in the gene encoding Klotho, a protein linked to longevity, have been found to induce premature aging, accompanied by hyperphosphatemia [71]. Klotho functions as a co-receptor for FGF-23, a key regulator of phosphate metabolism, and Klotho-deficient mice exhibit hallmarks of aging, including vascular calcification and arteriosclerosis. Interestingly, administration of secreted Klotho protein has been shown to inhibit renal fibrosis in murine models [72]. Following various renal insults, Klotho expression diminishes [73], and in CKD, its persistent downregulation is largely mediated by epigenetic mechanisms [74]. For instance, the uremic toxin indoxyl sulfate induces DNA methylation at the Klotho gene locus via oxidative stress and NF-κB activation, leading to Klotho suppression and accelerated cellular senescence [75]. Unraveling the epigenetic drivers of Klotho expression and identifying primary causative factors could lead to novel preventive or therapeutic interventions for kidney disease.

The accumulation of senescent cells is closely linked to organismal aging, as normal human cells undergo a finite number of divisions. This limitation results from telomere shortening in the absence of telomerase activity, triggering DNA damage and activating the p53-p21 and p16^INK4a^-retinoblastoma (Rb) pathways, ultimately leading to irreversible cell cycle arrest [76]. In the kidney, accumulation of p16^INK4a^-positive cells serves as a molecular hallmark of renal senescence. Epigenetic regulation plays a role in this process, particularly through histone H3K4 tri-methylation mediated by the MLL1/WDR5 complex. Senescence can be induced not only by chronological aging but also by stress events, such as AKI, which contribute to CKD progression through stress-induced senescence. Our research has demonstrated that the inhibition of MLL1 and WDR5—via MM-102 and OICR-9429, respectively—as well as siRNA targeting MLL1/WDR5 complex, reduces p16^INK4a^ expression and H3K4 tri-methylation in a mouse model of IRI-induced renal fibrosis. This, in turn, attenuates renal senescence, inflammation, and fibrosis [77]. These findings establish an epigenetic link between the AKI-to-CKD transition and stress-induced renal senescence.

Although no studies have specifically investigated senescence and epigenetic memory in nephrology, insights from other fields suggest that they are potentially related. For example, in aging mouse brains, dysregulation of H4K12 acetylation (H4K12ac) has been shown to cause age-related memory impairment, suggesting that H4K12ac may function as a key signal in memory formation [78]. Focusing on this area may open up new avenues for therapeutic development in kidney disease.

### Targeting hypertension

As blood pressure increases, so does the burden on the kidneys, further accelerating the decline in renal function. While salt intake typically raises blood pressure, environmental factors such as aging and stress also influence salt sensitivity. Consequently, hypertension is considered a disease in which environmental factors can directly impact gene function through epigenetic mechanisms. A few reports have detailed the involvement of epigenetics in hypertension. In a salt-sensitive animal model, the Dahl rat, salt intake has been shown to induce DNA methylation changes [79–81]. Salt loading has also been shown to promote salt-dependent hypertension through β2-adrenergic receptor (β2AR) stimulation, which cyclic AMP-dependently regulates histone deacetylase 8 (HDAC8) activity, thereby controlling WNK4 transcription to modulate Na^+^-Cl^−^ cotransporter (NCC) activity [4]. Furthermore, obesity-related hypertension in high-fat diet-fed mice is associated with increased HDAC1 levels and histone H3 acetylation, and administration of the HDAC inhibitor valproic acid was shown to suppress the progression of hypertension [82]. In aging mice, DNA methylation of the promoter region of *Klotho* reduces circulating soluble Klotho levels. Under salt-loading conditions, this epigenetic alteration activates the Wnt5a-RhoA pathway, contributing to age-related salt-sensitive hypertension [83]. In humans, dietary sodium intake has been shown to influence the expression of the histone demethylase enzyme LSD1 [84].

A phenomenon termed “salt memory” has been reported: salt-sensitive rats fed a high-salt diet and subsequently returned to a normal diet continued to experience elevated blood pressure for 3 months after reverting to the normal diet [85]. The involvement of epigenetic factors in the phenomenon of “salt memory” has been reported, with evidence suggesting a possible correlation between DNA methylation levels in cardiac tissue and the effects of salt memory [86]. However, the detailed mechanisms underlying this phenomenon remain unclear, highlighting the need for further research in this area.

## Perspective and conclusion

Given the reversibility of epigenetic regulation, novel approaches that enable gene-specific epigenetic control are emerging as promising therapeutic targets. Moreover, the reversal of epigenetic marks holds potential for treating chronic diseases characterized by persistent “epigenetic memory” [3].

Currently, clinical research on epigenetic drugs for particular disease targets is advancing. In 2006, vorinostat, the first HDAC inhibitor, was approved in the United States for the treatment of cutaneous T-cell lymphoma, followed by the approval of other HDAC inhibitors, including romidepsin, belinostat, and panobinostat, as anticancer agents in the United States, Europe, and Japan. Clinical trials for inhibitors of EZH2 and DOT1L have also been reported [87], and similar approaches could be applied to kidney disease. However, the long-term use of epigenetic drugs may carry the risk of cytotoxicity, potentially causing severe side effects in patients [88]. This underscores the need for further development of more effective and less toxic epigenetic drugs, such as those derived from natural products or medicinal plants, to provide safer therapeutic options.
